# Human herpesvirus-6 in hematopoietic stem cell transplant recipients: a prospective cohort study in Egypt

**DOI:** 10.1186/s12985-023-01980-w

**Published:** 2023-02-04

**Authors:** May Moheb Eldin Raouf, Nancy Mohammed Ouf, Manal Abdel Sattar Elsorady, Faika Mahmoud Ghoneim

**Affiliations:** 1 Medical Microbiology and Immunology Department, Faculty of Medicine, Alexandria University, 0 Khartoum Square, Azarita, Alexandria, Egypt; 2 Internal Medicine Department, Faculty of Medicine, Alexandria University, Alexandria, Egypt

**Keywords:** Human herpesvirus 6, Autologous, Allogeneic, Hematopoietic stem cell transplantation, HSCT, HHV-6 reactivation

## Abstract

**Background:**

Immunocompromised patients face reactivation of latent viruses that increase the risk of morbidity.

**Aim:**

The study aimed to detect human herpes virus 6 (HHV-6) reactivation among allogeneic (allo) and autologous (auto) hematopoietic stem cell transplant (HSCT) recipients and to correlate potentially attributed clinical manifestations to HHV-6 DNA plasma level.

**Methods:**

A prospective study included all (forty) patients undergoing allo and auto-HSCT from Jan 2020 till June 2022. Plasma samples were collected for HHV-6 serology, and for HHV-6 quantitative PCR at post-transplantation weeks 2, 4, 6. Demographic and clinical data were recorded.

**Results:**

Out of 40 peripheral blood stem cell transplant (PBSCT) recipients, 34 (85%) were HHV-6 IgG positive pre-HSCT. Of which, fourteen patients (14/34, 41.2%) showed positive HHV-6 DNaemia. HHV-6 DNAemia (15/40, 37.5%) was significantly higher among allo (8/12, 66.7%) versus auto (7/28, 25%) HSCT recipients (*p* = 0.030). Patients with HHV-6 DNAemia developed fever, delayed engraftment and bone marrow suppression in 6/15, 40%, thrombocytopenia (5/15, 33.3%), rash and pneumonitis (2/15, 13.3%), acute GVHD (aGVHD) (1/15, 6.7%). HHV-6 DNAemia ranged from 101 to 102,000 copies/mL. Univariate analysis identified conditioning with busulfan–cyclophosphamide as a significant risk (*p* = 0.043), while receiving BEAM protocol was a protective factor (*p* = 0.045). In multivariate analysis, receiving BEAM protocol retained significance (*p* = 0.040).

**Conclusion:**

Frequent HHV-6 reactivation was detected after HSCT, especially in allo-HSCT recipients with clinical manifestations which could not be otherwise explained. To our best knowledge this is the first study of HHV6 reactivation in HSCT recipients from Egypt. Raising awareness for HHV-6 reactivation manifestations and screening in HSCT recipients could be lifesaving.

## Introduction

Viral infection or reactivation increases morbidity in hematopoietic stem cell transplant (HSCT) recipients, particularly after allogeneic transplantation including herpes simplex virus (HSV), varicella zoster virus (VZV), cytomegalovirus (CMV), Epstein Barr virus (EBV) and human herpes virus 6 (HHV-6) [1, 2]. HHV-6 is part of to the β-herpesvirus subfamily. HHV-6 has been classified into two discrete species: HHV-6A and HHV-6B [3, 4]. HHV-6B is ubiquitous infecting 90% of humans early in life [5]. It is widely accepted that HHV-6B is the primary cause of exanthem subitum (roseola infantum or sixth disease) in children, whereafter it can establish latency [6]. HHV-6 establishes latency in CD34 positive hematopoietic cells, such as monocytes, macrophages, bone marrow progenitors and T-cells [7, 8]. Reactivation of latent HHV-6 may occur under immunosuppressive conditions. HHV-6 reactivation in HSCT recipients range from being asymptomatic to development of fever, skin rash, pneumonitis, myelosuppression, delayed engraftment, CMV reactivation, life threatening conditions as acute graft-versus-host disease (aGVHD) [9–12]. Moreover, HHV6 reactivation is the number one cause of encephalitis in transplant patients [13].

Polymerase chain reaction (PCR) has been considered as the pillar to detect HHV-6 reactivation. Nevertheless, it’s important to interpret the results in the settings of clinical disease [14, 15]. As HHV6 antiviral prophylaxis and treatment protocols are still of uncertain value [4, 11], and as we do not have national reports or previous studies that have addressed this issue, our study aimed to explore the magnitude of HHV6 reactivation in one of our HSCT transplant units. Data collected from multi centers would help experts to develop guidelines that can be lifesaving and increase the procedure success rates.

## Subjects and methods

### Study setting

A prospective cohort study was carried out at the Bone marrow transplant unit between January 2020 and June 2022. All patients undergoing allo and auto-HSCT for hematologic and lymphatic malignancies were included in the study.

### Data collection and clinical assessment

The following data were collected for all patients included in the study:

- *Demographic data* sex, age
- *Medical history* underlying disease indicating HSCT and transplantation regimens were recorded. According to the protocol of the bone marrow transplant unit, peripheral blood stem cell transplant (PBSCT) was used for all patients. Myeloablative conditioning **(**MAC) regimen received was busulfan 4 mg/kg/day PO plus cyclophosphamide 30 mg/kg/day (Bu–Cy) for 4 days for acute leukemia (cumulative dose 120 mg/kg total dose). Reduced intensity conditioning (RIC) regimens received were cyclophosphamide 50 mg/kg/day plus fludarabine 30 mg/m^2^/day for aplastic anemia, BEAM protocol (bendumustin 200 mg/m^2^/day, etoposide 200 mg/m^2^/day, AraC 400 mg/m^2^/day, melphalan 140 mg/m^2^/day) for lymphoma and melphalan 100 mg/m^2^/day for myeloma. All allogeneic patients received PBSCT from an HLA-identical sibling and received Methotrexate at post-transplant days 1, 3, 6, 11 as a mini-MTX GvHD prophylaxis (15 mg/kg/day at D1, followed by 10 mg/kg/day at D3, 6, 11. Prophylaxis against infections started before transplantation and continued up to 100 days post-transplant. All patients received acyclovir 1500 mg/m^2^/day for antiviral prophylaxis, levofloxacin 10 mg/kg/day for antibacterial prophylaxis, sulfamethoxazole-trimethoprim 5 mg/kg/day for prophylaxis against Toxoplasma gondii and Pneumocystis jirovecii and fluconazole 6 mg/kg/day for antifungal prophylaxis. Neutropenic fever work-up was carried out according to the IDSA guidelines [16].

*Clinical assessment* All patients were monitored for signs and symptoms for at least 6 weeks for symptoms that may be potentially related to HHV-6 as fever lasting for more than 2 days with no documented positive microbiological findings, skin rash without confirmed GVHD, delayed engraftment, thrombocytopenia or bone marrow suppression after engraftment, pneumonitis and CNS manifestations.

### Sample collection

Blood samples was aseptically withdrawn from each patient before transplantation and at post-transplantation weeks 2, 4 and 6. An additional sample was collected at post-transplantation week 8 in case HHV-6 DNAemia was detected at post-transplantation week 6. Centrifugation was done and samples were aliquoted into three labeled sterile eppendorf tubes and stored at – 70 °C.

### Serology

The HHV-6 serostatus of HSCT recipients was determined in plasma during the pretransplantation period using HHV-6 IgG ELISA (bioaasay technology laboratory, Shanghai) according to manufacturer 's instructions [17].

### Molecular detection of HHV-6 DNAemia

A total of 105 plasma samples were subjected to Real-time PCR testing. DNA was extracted using (Thermo Fisher Scientific GeneJET Viral DNA and RNA Purification Kit #K0821, Vilnius, Lithuania)^.^ RT-PCR amplification was performed with a thermal cycler (Rotor-Gene Q MDx) using forward primer sequence 5′ ACC CGA GAG ATG ATT TTG CG 3′ and reverse primer 5′ GCA GAA GAC AGC AGC GAG AT 3′ as previously described [18]. The human glyceraldehyde-3-phosphate dehydrogenase (GAPDH) gene was used as an internal control. All the primers used were synthesized by (Thermo Fisher Scientific, Invitrogen, UK).

The specificity of PCR products was verified using 2% agarose gel electrophoresis.

The quantification of HHV-6 viral load was done using a known amount of HHV-6 DNA that was taken in tenfold serial dilutions from 10^5^ to 10^1^ copies of HHV-6 genome to create a standard curve in the qPCR assay [19]. High level of HHV-6 copies was defined, based on previous findings, as plasma HHV-6 DNA ≥ 10.^4^ copies/mL [13, 20].

### Statistical analysis

Data were analyzed using IBM SPSS software package version 20.0 (Armonk, NY: IBM Corp) [21]. Qualitative and quantitative data were described using statistical parameters. Kaplan–Meier method was used for calculating the cumulative incidence of HHV-6 DNAemia. Logistic regression was done using univariate analysis, followed by multivariate analysis, where only factors with *p* values < 0.10 were included. *p* values < 0.05 were considered statistically significant.

### Ethics approval

The study was approved by the Ethics Committee, faculty of Medicine, Alexandria University.

## Results

### Patients’ characteristics

Male to female ratio was 5:3. The median age was 39.5 years (range, 19–72 years). Twelve patients received allo-HSCT and 28 patients received auto-HSCT. Allo-HSCT recipients included 7 acute myeloid leukemia (AML), 3 acute lymphocytic leukemia (ALL), one aplastic anaemia and one biphenotypic leukemia patients. Auto-HSCT recipients included 17 multiple myeloma, 7 Hodgkin’s lymphoma (HL) and 4 non Hodgkin’s lymphoma (NHL) patients. All patients received PBSCT (Table 1).

**Table 1:** Characteristics of allogeneic and autologous hematopoietic stem cell transplant recipients

	Allogeneic HSCT recipients (n = 12)		Autologous HSCT recipients (n = 28)	
	No	%	No	%
Gender				
Male	10	83.3	15	53.6
Female	2	16.7	13	46.4
Age (years)				
Min.–Max	19.0–43.0		19.0–72.0	
Mean ± SD	27.83 ± 7.36		45.89 ± 13.71	
Median (IQR)	27.50 (22–32)		45.0 (38–56)	
Source of stem cells				
PBSCT	12	100.0	28	100.0
Underlying haematologic disease				
AML	7	58.3	0	0.0
ALL	3	25.0	0	0.0
Biphenotypic acute leukemia	1	8.3	0	0.0
Aplastic anaemia	1	8.3	0	0.0
HL	0	0.0	7	25.0
NHL	0	0.0	4	14.3
Multiple Myeloma	0	0.0	17	60.7
HLA disparity				
Matched related	12	100.0		
*Conditioning regimen*				
MAC				
Busulfan + cyclophosphamide	11	91.7	0	0.0
RIC				
Cylophosphamide + fludarabine	1	8.3	0	0.0
BEAM protocol	0	0.0	11	39.3
Melphalan	0	0.0	17	60.7
GVHD prophylaxis				
Methotrexate + cyclosporine A	12	100.0		
Anti-viral prophylaxis				
Acyclovir	12	100.0	28	100.0
Seropositive (IgG)				
HHV-6	11	91.7	23	82.1
CMV	12	100.0	26	92.9
EBV	12	100.0	25	89.3

*HSCT* hematopoietic stem cell transplant, *PBSCT* peripheral blood stem cell transplant, *BMT* bone marrow transplant, *CBT* cord blood transplant, *AML* acute myeloid leukemia, *ALL* acute lymphocytic leukemia, *HL* Hodgkin’s lymphoma, *NHL* non Hodgkin’s lymphoma, *MAC* myeloablative conditioning, *RIC* reduced intensity conditioning, *GVHD* graft versus host disease, *HHV-6* human herpesvirus-6, *CMV* cytomegalovirus, *EBV* Epstein Barr virus

#### HHV-6 IgG testing

Thirty four HSCT recipients (85%) were positive for HHV-6 IgG. They included 11/12 allo and 23/28 auto-HSCT recipients. The difference between allo and auto-HSCT recipients in HHV-6 seopositivity was not statistically significant (χ^2^ = 0.598, *p* = 0.648).

### Detection of HHV-6DNA in plasma by real time PCR

**Fig. 1:**
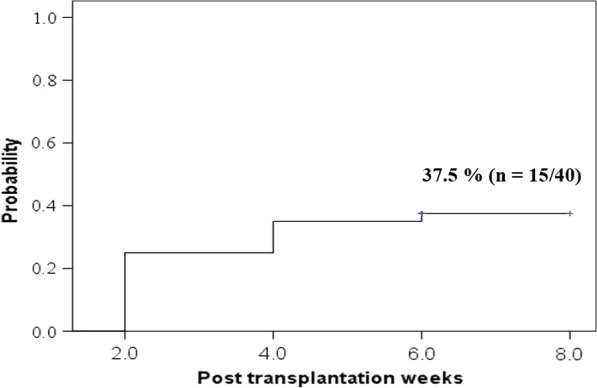
Cumulative incidence for first detection of HHV-6 DNAemia in plasma after HSCT using Kaplan–Meier curve

A total of 105 blood samples were obtained from the 40 HSCT recipients (equivalent to 40, 37, 26, 2 samples obtained at post transplantation weeks 2, 4, 6, 8 respectively). Not all scheduled samples were obtained because of 3 deaths or due to patients wellbeing and discharge. The onset and ending of an episode of HHV-6 DNAemia was determined by the first positive and first negative results in the corresponding PCR assay. Fifteen patients (37.5%) showed positive HHV-6 DNaemia in at least one sample (Fig. 1). Of which 14 were HHV-6 seopositive pretransplant. HHV-6 DNA was detected in 8 allo (8/12, 66.7%) and 7 auto-HSCT recipients (7/28, 25%) (χ^2^ = 6.222, *p* = 0.030). Out of 105 plasma samples submitted for RT-PCR testing, 20 (19%) were HHV-6 DNA positive. Ten patients with HHV-6 DNAemia (10/15, 66.7%) had only one positive sample and 5 (33.3%) had 2 positive samples. The median duration of HHV-6 DNAemia was 14 days (range, 14–28 days).

### HHV-6 viral load

**Fig. 2:**
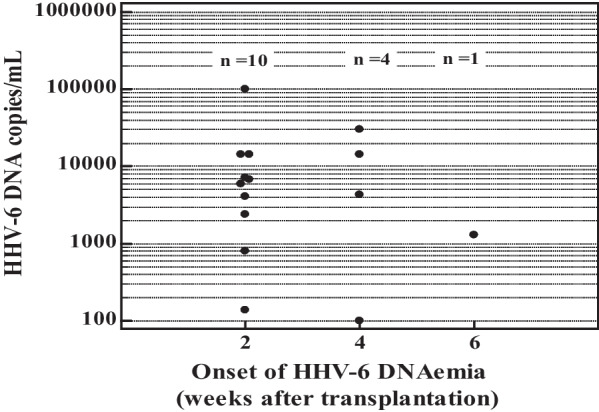
The relation between the onset of HHV-6 DNAemia and viral load for patients positive for HHV-6 DNA (n = 15)

Quantitation of HHV-6 DNAemia showed a range of 101 to 102,000 copies/mL plasma. The median was 5990 copies/mL. The relationship between the onset of HHV-6 DNAemia and HHV-6 viral load was assessed. Ten HHV-6 DNA–positive cases (10/15, 66.6%) and 3 cases (3/5, 60%) with high HHV-6 DNA copies (≥ 10,000 copies/mL) were distributed at post-transplantation week 2. (Fig. 2).

### Factors affecting HHV6 reactivation

Univariate analysis identified conditioning with MAC (Busulfan and Cyclophosphamide) as a significant risk factor for HHV-6 reactivation (OR:4.594, 95% CI 1.052–20.057, *p* = 0.043), while receiving BEAM protocol was a protective factor (OR: 0.107, 95% CI 0.012–0.949, *p* = 0.045). Nevertheless, in the multivariate analysis, only receiving the BEAM protocol retained significance (OR: 0.054, 95% CI 0.003–0.877, *p* = 0.040) (Table 2).

**Table 2:** Univariate and multivariate analysis for assessing human herpesvirus-6 reactivation factors

	Univariate		Multivariate^#^	
	*p*	OR (95% C.I)	*p*	OR (95% C.I)
Age (years)	0.471	0.984 (0.940–1.029)		
Gender^c^	0.085	0.271 (0.061–1.200)	0.170	0.261 (0.038–1.779)
Donor sex^c^	0.471	0.333 (0.023–4.736)		
Underlying haematologic disease^c^				
AML^c^	0.248	2.667 (0.506–14.063)		
ALL^c^	0.305	3.692 (0.305–44.692)		
Biphenotypic acute leukemia^c^	0.999	–		
Aplastic anaemia^c^	0.999	–		
HL^c^	0.191	0.226 (0.024–2.097)		
NHL^c^	0.999	–		
Multiple Myeloma^c^	0.804	0.848 (0.231–3.114)		
*Conditioning regimen*				
MAC				
Busulfan + cyclophosphamide^c^	0.043*	4.594* (1.052–20.057)	0.474	1.915 (0.324–11.328)
RIC				
Cyclophosphamide + fludarabine^c^	1.000	–		
BEAM protocol^c^	0.045*	0.107* (0.012–0.949)	0.040*	0.054* (0.003–0.877)
Melphalan^c^	0.804	0.848 (0.231–3.114)		
Pre-transplant HHV-6 IgG^c^	0.276	3.50 (0.368–33.308)		
Unexplained fever^c^	0.463	0.615 (0.168–2.252)		
Cutaneous rash^c^	0.999			
Thrombocytopenia^c^	0.057	5.750 (0.950–34.787)	0.800	0.584 (0.009–38.014)
Delayed engraftment/bone marrow suppression^c^	0.099	3.500 (0.791–15.479)	0.170	13.239 (0.332–528.126)
Days to platelets engraftment (mean)	0.140	0.794 (0.585–1.078)		
Days to neutrophils engraftment (mean)	0.458	1.064 (0.903–1.253)		
aGVHD^c^	1.000	–		

*HSCT* hematopoietic stem cell transplant, *AML* acute myeloid leukemia, *ALL* acute lymphocytic leukemia, *HL* Hodgkin’s lymphoma, *NHL* non Hodgkin’s lymphoma, *MAC* myeloablative conditioning regimen, *RIC* reduced intensity conditioning, *HHV-6* human herpesvirus-6, *aGVHD* acute graft versus host disease, *OR* odd’s ratio, *C.I*. confidence interval

^c^Categories

^#^All variables with *p* < 0.1 was included in the multivariate

*Statistically significant at *p* ≤ 0.05

### HHV-6-associated symptoms, biological events and outcomes

Out of 15 HHV-6 DNA positive patients, 2 (13.3%) were asymptomatic, and the rest developed symptoms or biological effects with no other identifiable explanatory causes. These included fever (40%), delayed engraftment and bone marrow suppression (40%), thrombocytopenia (33.3%), rash (13.3%), pneumonitis (13.3%) and aGVHD (6.7%), none of the patients developed encephalitis. Although the incidence of most of these symptoms was higher among HHV-6 DNA positive patients compared to those negative for HHV-6 DNA, none of them reached statistical significance. Median time to platelet and neutrophil engraftment in patients with HHV-6 DNAemia was 11 and 12 days, respectively. HHV6 reactivation had no significant impact on time to platelet and neutrophil engraftment (Table 3).

**Table 3:** Descriptive analysis for clinical and biological manifestations potentially related to HHV-6 reactivation

	Patients negative for HHV-6 DNAemia (n = 25)		Patients positive for HHV-6 DNAemia (n = 15)	
	No	%	No	%
Asymptomatic	6	24.0	2	13.3
Unexplained fever	13	52.0	6	40.0
Cutaneous rash	0	0.0	2	13.3
Pneumonitis	2	8.0	2	13.3
aGVHD	0	0.0	1	6.7
Encephalitis	0	0.0	0	0.0
Thrombocytopenia	2	8.0	5	33.3
Delayed engraftment/bone marrow suppression	4	16.0	6	40.0
Days to platelets engraftment				
Min.–Max	5.0–22.0		8.0–13.0	
Mean ± SD	12.16 ± 3.14		10.80 ± 1.61	
Median (IQR)	12.0 (11.0–14.0)		11.0 (10.0–12.0)	
Days to neutrophils engraftment				
Min.–Max	7.0–24.0		9.0–28.0	
Mean ± SD	12.84 ± 3.06		13.80 ± 5.16	
Median (IQR)	12.0 (11.0–13.0)		12.0 (10.0–15.50)	

*HHV-6* human herpes virus-6, *aGVHD* acute graft versus host disease

The detected clinical and biological manifestations were not related statistically to HHV-6 viral load (Table 4).

**Table 4:** The relation between HHV-6 viral load and clinical or biological manifestations potentially related to HHV-6 in HHV-6 DNAemia positive patients

	Viral load				Test of significance	*p*
	< 10^4^ (n = 10)		> 10^4^ (n = 5)			
	No	%	No	%		
Unexplained fever	5	50.0	1	20.0	χ^2^ = 1.250	^FE^p = 0.580
Cutaneous rash	0	0.0	2	40.0	χ^2^ = 4.615	^FE^p = 0.095
Pneumonitis	0	0.0	2	40.0	χ^2^ = 4.615	^FE^p = 0.095
aGVHD	1	10.0	0	0.0	χ^2^ = 0.536	^FE^p = 1.000
Thrombocytopenia	3	30.0	2	40.0	χ^2^ = 0.150	^FE^p = 1.000
Delayed engraftment/bone marrow suppression	4	40.0	2	40.0	χ^2^ = 0.000	^FE^p = 1.000
Days to platelets engraftment (mean)						
Mean ± SD.(Range)	10.60 ± 1.58 (8.0–13.0)		11.20 ± 1.79 (9.0–13.0)		t = 0.666	0.517
Median (IQR)	10.50 (10–11)		11.0 (10–13)			
Days to neutrophils engraftment (mean)						
Mean ± SD.(Range)	14.80 ± 5.90 (10.0–28.0)		11.80 ± 2.68 (9.0–16.0)		t = 1.067	0.305
Median (IQR)	13.50 (10–17)		12.0 (10–12)			

*HHV-6* human herpes virus-6, *aGVHD* acute graft versus host disease, *CMV* cytomegalovirus

χ^2^: Chi square test, FE: Fisher Exact, t: Student t-test

*p*: *p* value for comparing between negative and positive HHV-6 DNAemia

In all patients with HHV-6 DNAemia, the viremia resolved spontaneously on the unit standard protocol without specific treatment.

## Discussion

Reactivation of HHV-6 is noticed after HSCT, but the significance of this reactivation clinically, is still not fully clarified. The present study was made to assess the incidence of HHV-6 reactivation among allo and auto-HSCT recipients and the relationship between this reactivation and developing complications after HSCT.

In this study, HHV-6 IgG was detected in 85% of HSCT recipients pre-transplant. This was nearly similar to Chapenko et al*.* who declared that 81.8% of patients were HHV-6 seropositive [22]. While, Yoshikawa et al*.* found that all the study participants were seropositive to HHV-6 pre-HSCT [23]. No significant difference was noted in our study between allo and auto-HSCT recipients regarding seropositivity to HHV-6 (*p*** = **0.648). Similar results were found by Imbert-Marcille et al.[24]

In the present study, 37.5% of HSCT recipients showed positive HHV-6 DNaemia after transplantation. A statistically significant difference regarding HHV-6 DNAaemia was detected allo- versus auto-HSCT (66.6% vs 25%, *p* = 0.030). The immunodeficiency state that occurs in auto-HSCT recipients is usually less persistent than in allo-HSCT patients (secondary to preconditioning and immunosuppressant drugs for GVHD prophylaxis). Subsequently, auto-HSCT recipients are generally considered less susceptible to viral reactivation than allo-HSCT recipients. Our results were very similar to *Yoshikawa* et al*.* who demonstrated that 37.8% of HSCT recipients were positive for HHV-6 DNAemia, and that the incidence was significantly greater among allo-HSCT recipients than among auto-HSCT recipients [25]. Miyoshi et al. reported similar results concerning allo-HSCT (68%) but with higher Figs. (61%) among auto-HSCT [26]. Imbert-Marcille et al. reported 42.5% HHV-6 reactivation among both groups [24]. Some studies reported lower reactivation rates among allo-HSCT recipients (35%, 47.2%, 50%, 58.5%) [3, 27–29] and among auto-HSCT recipients (9% and 11.4%) [12, 30]. Other studies demonstrated higher rates among allo-HSCT recipients (78%) [31] and among auto-HSCT recipients (41.7%, 72%) [32, 33]. Reasons for these different results may depend on the nature of the underlying diseases and source of stem cells received whether PBSCT, bone marrow transplant (BMT) or cord blood transplant (CBT). Another explanation could be the variations in the clinical specimens being tested for HHV-6 DNA (PBMCs, serum or plasma), as latent HHV-6 DNA can be detected in PBMCs by PCR, resulting in false-positive results [15]. Thus, in the current study, plasma samples was used as plasma is cell-free, to avoid the detection of latent HHV-6 infection. Also, the presence of HLA mismatches could help in HHV-6 reactivation as identified in some studies [20, 34]. Here, none of the allo-HSCT recipients received transplant from HLA mismatched donors. Also, many studies demonstrated that HHV-6 DNA was more frequently detected in recipients of CBT than in recipients of BMT or PBSCT [20, 35]. This may be explained by the fact that most of the T cells in cord blood are naeive T cells and do not contain memory T cells against the virus.

Around 93% of the HHV-6 DNA positive cases in the current study were detected between post-transplantation weeks 2 and 4. Similar results was demonstrated by many studies [24, 25, 36] Ogata et al. detected HHV-6 DNA in plasma most frequently at 15–21 days after HSCT [13].

In the present study, HHV-6 plasma viral load ranged from 101 to 102,000 copies/ml. The median was 5990 copies/mL, and 60% (3/5) of samples with high HHV-6 DNA copy numbers were distributed at post-transplantation week 2. Yamane et al. reported that in patients with HHV-6 DNAemia, the number of HHV-6 DNA copies ranged from 200 to 200,000/mL of plasma and 80% of samples with high HHV-6 DNA copies were distributed between weeks 2 and 4 after transplantation [20].

No significant relation was detected between HHV-6 plasma viral load and the severity of symptoms or the patient outcomes in this study. This was similar to Dulery et al. [37] nevertheless, high HHV-6 viral load was illustrated to be significantly associated with delayed platelet engraftment and bone marrow suppression, aGVHD*,* and encephalitis in other studies [27, 31, 38].

The associations of different risk factors for HHV-6 reactivation were studied in the current study. Regarding demographic data, age, sex and sex mismatch between HSCT donor and recipient had no effect on HHV-6 reactivation after transplant. Age had no effect in many studies [3, 7, 20], however, Zerr et al. found that younger age was significantly associated with HHV-6 reactivation [39]. Male gender was also not significantly related to HHV-6 reactivation in many studies [3, 20, 25], while Ogata et al. and Jeulin et al. found a significant association between male gender and HHV-6 DNAemia after HSCT [13, 40]. Dulery et al. reported no relation between sex-mismatch and the incidence of HHV-6 reactivation [37]. in contrast, Zerr et al. reported that sex-mismatched graft was significantly associated with HHV-6 reactivation [39].

Serologically, no significant effect for pre-transplant HHV-6 IgG on HHV-6 reactivation was found (*p* = 0.276). Nakayama et al. reported association of low pre-transplant HHV6 IgG and HHV6 reactivation in Cord blood recipients but not in PBSCT recipients [3]. In our study, only one patient was seronegative for HHV-6 IgG before transplantation, and developed HHV-6 DNAemia at the 2nd week post-transplantation. We suggest that transmission of HHV-6 may have occurred through donor transfused blood cells, similar to what was speculated in previous studies [20, 41].

Regarding the effect of the conditioning regimen, the univariate analysis identified that receiving Busulfan and Cyclophosphamide as MAC was a significant risk for development of HHV-6 DNAemia after transplantation (*p* = 0.043), while receiving BEAM RIC protocol was a protective factor (*p* = 0.045). In the multivariate analysis, receiving BEAM protocol retained significance (*p* = 0.040). Our findings may support the higher myeloablative and immunosuppressive intensity of Busulfan and Cyclophosphamide regimen compared to BEAM protocol [42]. Dulery et al. and Jeulin et al. reported that MAC was a risk factor for HHV-6 reactivation [37, 40], While, Nakayama et al. and Iesato et al. didn’t find a significant association between the conditioning regimen received and HHV-6 reactivation [3, 43].

In a study on the same patient group, CMV DNAemia was detected in 13/40 (32.5%) of HSCT recipients at a median of 6 weeks post-transplantation. CMV reactivation occurred in 3/12 (25%) of allo-HSCT recipients, and 10/28 (35.7%) of auto-HSCT recipients with no statistically significant difference found (*p* = 0.716) [44].

Our results showed that 13.3% of HHV-6 DNAemia positive patients were asymptomatic. A wide range of asymptomatic HHV-6 reactivation rates (5% and 41.9%) have been reported Dulery et al. and Hentrich et al. respectively [38, 42]. The development of symptoms that may be attributed to HHV-6 was illustrated in our study, and was compared with patients who were negative for HHV-6 DNAemia. None of the symptoms among HHV-6 DNA positive patients reached statistical significance. Unexplained fever was detected in 40% of HHV-6 DNA positive patients. Shargian-Alon et al. reported 97% of patients had sustained fever, however, this was also insignificant [30]. On the contrary, Imbert-Marcille et al*.* illustrated that unexplained fever was significantly associated with HHV-6 reactivation [24]. Cutaneous rash occurred in 13.3% of HHV-6 DNA positive patients in our study. This was coherent with some studies that found no significant relation [24, 30, 38]. In contrast, Yoshikawa et al*.* reported that rash was significantly associated with HHV-6 viremia after HSCT [25]. Pneumonitis was observed in 13.3% of patients with HHV-6 DNAemia in our study. This was consistent with Shargian-Alon et al., Yoshikawa et al. and Hentrich et al. who also reported the relation as insignificant [25, 30, 38].

In the present study, delayed engraftment or bone marrow suppression occurred in 40% of patients positive for HHV-6 DNAemia and the median day of platelet and neutrophil engraftment were post-transplantation days 11 and 12 respectively. Similar results were reported by Lanza et al*.* where the engraftment occurred at median post-transplantation day 12 [45]. This was in agreement with various studies who demonstrated that neutrophil and platelet engraftment were not significantly affected by HHV-6 reactivation [25, 29, 38, 43]. On contrast, other studies reported a significant association between HHV-6 reactivation and bone marrow suppression or delayed engraftment particularly for platelets [24, 37, 39].

Only one patient out of the 15 HHV-6 DNA positive patients showed manifestations suspicious of aGVHD. Sashihara et al*.*, as well, couldn’t find a clear association between HHV-6 reactivation and aGVHD [35]. On the other hand, several studies found that active infection with HHV-6 was significantly associated with a high risk of aGVHD following HSCT [28, 29, 38, 43].

## Conclusion

HHV-6 reactivation is frequently detected after HSCT, especially in allo-HSCT recipients. HHV-6 reactivation could lead to higher morbidity in allo and auto-HSCT recipients. Patients showed multiple manifestations of HHV6 reactivation which could not be otherwise explained. All patients experiencing symptoms potentially attributed to HHV-6 early after transplantation, should be tested for HHV-6 DNAemia. To our knowledge this is the first study of HHV6 reactivation in HSCT recipients from Egypt. Raising Awareness for rapid screening and diagnosis may prevent excess work up, prolonged hospitalization and could be lifesaving.

Limitations of our study include the small number of patients as well as short period of follow up, as this was affected by the lockdown during the COVID-19 pandemic which necessitated downsizing of the number of HSCT procedures and short hospital stay. Combined national and global Multicenter studies are needed to assess the potential benefit of prophylactic and treatment protocols in active infection.

## Data Availability

Available upon reasonable request from the corresponding author.

## Code availability

Non applicable.
